# The Scaphoid Safe Zone: A Radiographic Simulation Study to Prevent Cortical Perforation Arising from Different Views

**DOI:** 10.1371/journal.pone.0170677

**Published:** 2017-01-23

**Authors:** Qi Quan, Lei Hong, Biao Chang, Ruoxi Liu, Yun Zhu, Jiang Peng, Qing Zhao, Shibi Lu

**Affiliations:** 1 Department of Orthopedic Surgery, Key Laboratory of Musculoskeletal Trauma &War Injuries PLA, Beijing Key Lab of Regenerative Medicine in Orthopedics, Chinese PLA General Hospital, Beijing, China; 2 Department of Orthopedic Surgery, First Affiliated Hospital of PLA General Hospital, Beijing, China; 3 School of the Biomedical of Sciences, Li Ka Shing Faculty of Medicine, The University of Hong Kong (HKU), Hong Kong, China; Harvard Medical School/BIDMC, UNITED STATES

## Abstract

**Purpose:**

The purpose of this study was to simulate and calculate the probability of iatrogenic perforation of the scaphoid cortical bone when internal fixation appeared to be safe on radiographs. The results will assist surgeons in determining proper screw placement.

**Methods:**

Thirty scaphoids were reconstructed using computed tomography data and image-processing software. Different central axes were determined by the software to simulate the surgical views. The safe zone (SZ) and risk zone (RZ) were identified on the axial projection radiographs by comparing the scaphoid bone stenosis measured by the fluoroscopic radiographs with a three-dimensional reconstruction of the scaphoid stenosis. Each original axial projection radiograph was zoomed and compiled to match a calculated average image. The RZ, SZ, and probability of perforations in various quadrants were calculated.

**Results:**

Using a volar view (approach), the mean risks of cortical perforation were 25% with screws and 36% with k-wires. Using a dorsal view (approach), the mean risks of cortical perforation were 18% with screws and 30% with k-wires. A high risk of perforation was detected at the ulnar–dorsal zone.

**Conclusion:**

Surgeons should be wary of screws that appear to lie close to the scaphoid cortex on both anteroposterior (AP) and lateral radiographs, particularly in the ulnar–dorsal and radial–dorsal quadrants, because such screws are likely to perforate the cortex. The position of the internal fixator should be assessed using a diagram outlining the various SZs. Therapeutic, Level III.

## Introduction

Scaphoid fractures account for 10% of all hand fractures[1] and almost 60% of all carpal fractures[2]. The typical mechanism of injury is a fall onto a hyperextended and radially deviated wrist. The rate of misdiagnosis of scaphoid fractures is as high as 30% with conventional radiography[3]. Because internal fixation provides reasonable results, both patients and surgeons are willing to use intraosseous screw fixation to treat minimally and acutely displaced scaphoid fractures[4]. The goal of such treatment in young adults is to prevent carpal collapse and degenerative arthritis.

Although many techniques are available for internal fixation of a scaphoid fracture, the nonunion rate of scaphoid fractures is up to 10%[5]. A main cause of non-union is damage to the dorsal artery. The aim of stable fixation of a scaphoid fracture is to minimize the risk of non-union and osteonecrosis. Therefore, quality of the fixation determines the prognosis of patients with a scaphoid fracture.

A screw that appears to lie very close to the joint surface on a radiograph may have actually perforated the femoral head while still appearing to be located within the head[6]. Similarly, if a screw is inserted too close to the scaphoid waist cortex, the screw may have also penetrated that portion of the waist. Furthermore, the position of a guidewire located perpendicular to anteroposterior (AP) radiographs cannot be determined accurately. Therefore, this study calculated the probability of iatrogenic perforation of the scaphoid cortical bone when screws/K-wires appear to be positioned satisfactorily on radiographs. These results will help surgeons identify safe positions when placing screws or K-wires within the scaphoid.

## Materials and Methods

### Materials

We analyzed computed data from the wrists of 30 adults (15 females and 15 males; mean age, 28; range, 19–47 years) who underwent scanning for a non-scaphoid disorder between November 2015 and January 2016. This study obtained written approval from the Ethics Committee of the Chinese PLA General Hospital, Beijing, China (Consent: No. 2015.304.GuoZiRan), and all participants provided written informed consent. All computed tomography (CT) scans were undertaken using an 0.8-mm slice CT scanner. Scanning was performed from the proximal interphalangeal joint to the distal one-third of the forearm. Data from all cases underwent processing, as described below.

### Methods

Before we discuss our simulation of the radiographs and analysis of the diagram, several concepts should be clarified. The safe zone (SZ) within the projected image was defined as the positions where the screws or K-wires were apparently and actually positioned inside the scaphoid on both radiographs. The risk zone (RZ) was defined as the position in which the screw or K-wires appeared to be within the scaphoid on both radiographs when it/they actually perforated the cortex. Perforation risk was defined as the ratio of RZ area.

In short, the core steps of our calculations are summarized as:

1. Radiographs of the scaphoid SZ (A false safety zone cause perforation.) under fluoroscopy were simulated.
2. Size of the scaphoid stenosis was determined by setting a boundary line tangential to the curve of the mid-scaphoid on different radiographs.
3. The overlapping zone was the SZ compared with the actual stenosis of the scaphoid (three-dimensional [3D] reconstructed model). Non-overlapping zones were RZs. These non-overlapping zones were not determined by AP or lateral radiographs during surgery.

### Simulated radiographs

**Fig 1:**
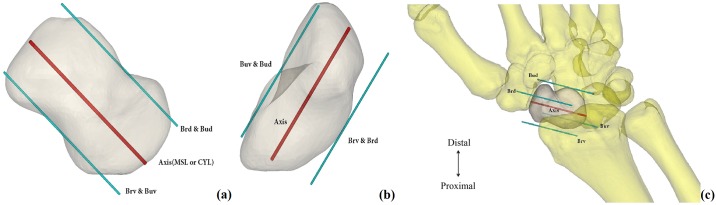
Simulated radiographs views and three-dimensional simulation of the reconstruction. (a), (b): Simulated radiographs anteroposterior (AP) and lateral views, Boundary radial–volar (Brv) line was parallel to the screw axis within the scaphoid was maximized (MSL)/cylinder (MSL/CYL) and lateral view, tangential to the mid- scaphoid curve. The radial–dorsal boundary (Brd), the ulnar–volar boundary (Buv), and the ulnar–dorsal boundary (Bud) were constructed and simulated using the same method. (c): Three-dimensional simulation of the reconstruction.

**Fig 2:**
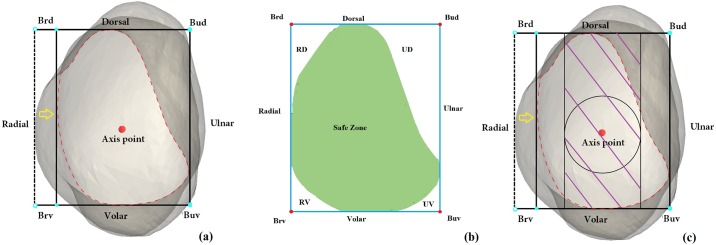
Projection views. (a) Axillary view. Original radial boundary (dotted line) was translated toward the ulnar side (yellow arrow). Red dot shows the axis point. (b) Axis projection view. Figure shows the risk zone (RZ), including the RD, UD, RV, and UV quadrants. The safe zone (SZ) is denoted by the green area. Blue lines mark the boundaries. (c) Screw cross-section view. Circles represent the cross-section of the screw, and purple areas represent the trajectory of the screw. We could not accurately identify the screw’s position when it was perpendicular on AP radiographs.

**Fig 3:**
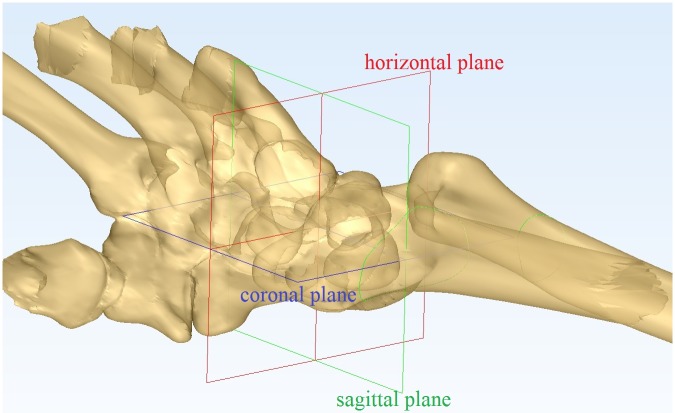
Wrist coordinate system. Horizontal plane(red), sagittal plane(green) and coronal plane(blue).

Mimics medical imaging software (Materialise, Leuven, Belgium) was used to analyzed the CT data and export the 3D models of the wrist as follows:

1. The following two methods were selected to establish the central axis of the scaphoid, and we calculated the scaphoid axis to prepare for dorsal or volar viewing.
  - The length of the screw axis within the scaphoid was maximized (MSL)[7]. To calculate the MSL, code was written to measure the longest axis that could be completely contained within the inner surface [7]. This code was uploaded to the support information (S1 Text). The axis for surgery was established using the volar percutaneous approach.
  - The axis for the best-fit cylinder (CYL) to the scaphoid was determined [7] using a least-squares algorithm (Geomagic Studio; Geomagic, Inc., Rock Hill, SC, USA). The screw axis was defined as the central axis of the resulting CYL[7]. This axis was established for surgery using the dorsal percutaneous approach.
2. The MSL or CYL lines were used to mark the scaphoid, and four blue border lines were drawn to establish the boundaries[8]. The boundary radial–volar (Brv) line was parallel to the MSL/CYL line and tangential to both curves of the scaphoid stenosis on the AP and lateral radiographs (Fig 1a and 1b). As shown in Fig 1c, we identified three other boundaries parallel to the axis using a similar method: the radial–dorsal boundary (Brd), the ulnar–volar boundary (Buv), and ulnar–dorsal boundary (Bud).
3. The surgical view was simulated to construct a projection along the axis line. The view was rotated until the boundary lines appeared as four dots (Brd, Bud, Brv, and Buv; Fig 2a). Because the anatomical features of the scaphoid on the radial side tend to bulge [9], the radial side of the border (Brd and Brv attachments) was moved toward the ulnar side because screw travel was too short in this part (S1 Fig). The screws or K-wires did not pierce the cortex of the radial bulge of the scaphoid. However, because screw travel was too short, the fracture fragments could not be fixed well. The result may be improved by correcting for the radial bulge of the scaphoid, but this would increase the difficulty of the actual surgery (S1 File). Therefore, we moved the radial side of the border toward the ulnar side until it was touching most of the accumulated bone cortex radial boundary, as shown on the axis view (Fig 2a). The diagram was then ready for analysis (Fig 2b).
4. The virtual screw was set (diameter, 2.7 mm). The ulnar and radial borders were moved to the central axis until they touched the virtual screw (Fig 2c). We set the virtual screw and moved the border because the diameter of the screw cannot be ignored compared with the size of the scaphoid and because the standard implant position to treat scaphoid fractures with screws can be as far as the central scaphoid. Setting the screw in the central part of the AP radiograph made it easier to succeed. Therefore, we hoped to remove the AP radiograph-induced perforation risk
5. To establish the wrist coordinate system, the two styloid processes of the forearm and the base of the third metacarpal bone were set as coordinate points, and the coronal plane was established using those three points. A horizontal plane, which was perpendicular to the center line of the radial shaft, was created through two of the styloid processes. The sagittal plane passed through and was perpendicular to the coronal and horizontal planes (S1 File). All these points are shown in Fig 3. (S2 Fig)

### Diagram analysis

The projection pictures were processed by MATLAB software (MATLAB Inc., Natick, MA, USA). The middle of the scaphoid project zone was defined as the SZ (Fig 2b, green). The RZ was marked by the following quadrants: RV, RD, UV, and UD (Fig 2b). A parallelogram was delineated by four dots (Brd, Bud, Brv, and Buv). Various data were calculated, including the area ratio RV/parallelogram area (PA) × 100%, RD/ PA × 100%, UV/ PA × 100%, and UD/ PA × 100%. The MSL and CYL axis were calculated separately. We programmed the algorithms, custom coded the data, and uploaded the computer program using “txt” format, and this information is as S2 Text. The calculations were based on calculus.

The angles between the axis and the sagittal, coronal, and horizontal planes were measured by Mimics.

### Statistical methods

SPSS Statistics ver. 22 (IBM Corp., Armonk, NY, USA) was used to analyze the data. We performed chi-square tests to analyze the effect of sex on variables of interest. P-values < 0.05 were considered significant. All values are presented as means and standard deviations (SDs).

## Results

**Fig 4:**
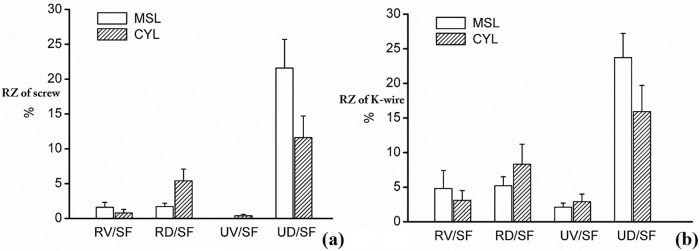
Mean ratio of different risk zones (RZs). (a): Mean ratio of different risk zones (RZs) with screw arising from the screw axis within the scaphoid was maximized (MSL)/cylinder (MSL/CYL) axis. (b): Mean ratio of different RZs with K-wire arising from the MSL/CYL axis.

All 30 scaphoids had RZs. The mean RZs from the MSL axis view were 24.9% (SD 1.7%) with screws and 36% (SD 3.5%) with k-wires. The mean RZs from the CYL axis view were 18.3% (SD 2.7%) with screws and 30.3% (SD 3.9%) with k-wires. A high risk of perforation was detected at the ulnar–dorsal zone (Fig 4). Details about the proportion of perforations are shown in Table 1 (S3 Text).

**Table 1:** Proportions of perforation.

Component	MSL axis view(volar view)		CYL axis view(dorsal view)	
	Screw	K-wires	Screw	K-wires
RV/SF· 100%	1.6% (SD 0.5%)	4.8% (SD 2.1%)	0.8% (SD 0.2%)	3.1% (SD 1.2%)
RD/SF· 100%	1.7% (SD 0.5%)	5.2% (SD 2.9%)	5.4% (SD 1.4%)	8.3% (SD 2.3%)
UV/SF· 100%	-----	2.1% (SD 1.0%)	0.4% (SD 0.2%)	2.9% (SD 1.0%)
UD/SF· 100%	21.6% (SD 1.7%)	23.7% (SD 3.5%)	11.6% (SD 2.4%)	15.9% (SD 3.8%)

The angles between the axis and the sagittal, horizontal, and coronal planes were approximately 30.2°(SD 3.95; range 23.8–38.7°), 33.2°(SD 4.82; range 27.2–47.9°), and 44.3°(SD 3.96;range 37.2–51.7°) in the volar axial view, and they were 25.7°(SD 1.89; range 23.2–30.6°), 41.1°(SD 2.47; range 37.1–47.8°), and 36.8°(SD 2.8; range 32.2–41.2°) in the dorsal axial view, respectively. The mean angles are shown in Tables 2 and 3 (S4 Text).

**Table 2:** Volar axial view of X-ray projection (MSL axis).

Component (degrees)	Male		Female		P
	Mean	Range	Mean	Range	
Angle projection to the sagittal plane	30.7 (SD4.44)	23.8–38.7	29.4 (SD3.42)	23.8–35.6	>0.05
Angle projection to the horizontal plane	33.5 (SD4.26)	27.8–39.8	32.9 (SD5.46)	27.2–47.9	>0.05
Angle projection to the coronal plane	44.5 (SD4.18)	37.2–51.2	44.1 (SD3.85)	38.7–49.8	>0.05

Sex’s p values are based on chi-square test

**Table 3:** Dorsal axial view of X-ray projection(CYL axis).

Component (degrees)	Male		Female		P
	Mean	Range	Mean	Range	
Angle projection to the sagittal plane	25.6 (SD1.75)	23.2–28.3	25.8 (SD2.06)	23.8–30.6	>0.05
Angle projection to the horizontal plane	40.6 (SD1.87)	37.1–43.5	41.6 (SD2.93)	37.2–47.8	>0.05
Angle projection to the coronal plane	37.4 (SD2.62)	33.6–40.5	36.1 (SD2.91)	32.2–41.2	>0.05

Sex’s p values are based on chi-square test

## Discussion

Inexperienced surgeons usually place the guide k-wire for a headless screw under the AP view. However, as the narrowest part of the scaphoid waist is not fully displayed on a radiograph, the SZ is exaggerated. Positioning the guide K-wire under the AP view is occasionally acceptable; however, a perforation of the cortical bone in the waist concavity is detected on the oblique or lateral view. This approach expends operation time and increases the patient’s radiation exposure.

### Ideal screw position

Several criteria should be met when fixating scaphoid screws or K-wires. The internal fixator should have sufficient length across the fracture fragments and be centered as far as possible to achieve the ideal screw position[10]. Many authors recommend stabilizing all scaphoid fractures by placing a screw along the central axis of the scaphoid, regardless of the fracture type [11–14]. Intraoperative C-arm fluoroscopic guidance remains the most common method to navigate while inserting screws. Unfortunately, two-dimensional images present severe limitations, regardless of imaging resolution, because, due to insufficient data, a screw may appear to be placed adequately on conventional radiographs when it is actually perforating the scaphoid waist cortex.

### C-arm fluoroscopic guidance to obtain the SZ view

**Fig 5:**
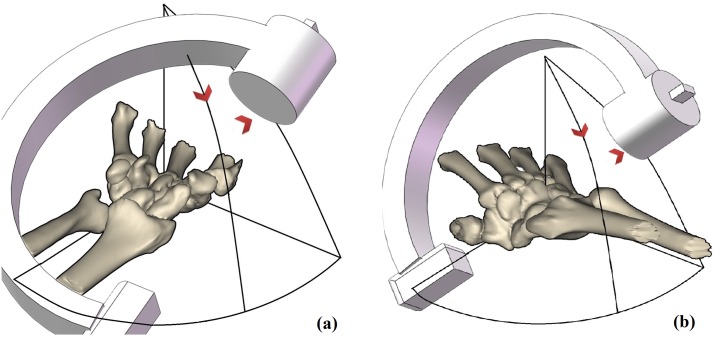
Axial view was obtained by tilting the C-arm of the fluoroscope. (a): Volar axial view of the X-ray projection (MSL axis). (b): Dorsal axial view of the X-ray projection (CYL axis).

Radiographs taken from different angles can help the surgeon determine the axial view of the scaphoid SZ on intraoperative fluoroscopy. The volar axial view can be obtained by tilting the C-arm of the fluoroscope by approximately 30° to the radial side from the sagittal plane and then rotating it approximately 45° to the coronal plane. The dorsal axial view can be obtained by tilting the C-arm by approximately 25° to the radial side from the sagittal plane and then rotating it approximately 35° to the coronal plane (Fig 5).

### Central axis of the scaphoid and axial view

There is no recognized method for determining the central axis of the scaphoid. However, as described previously, two approaches can be used to determine the axis [7]. In the first method, the length of the screw axis within the safe zone (MSL) is maximized to calculate the longest axis that can be completely contained within the inner surface of the scaphoid and is considered the most appropriate axis for the volar surgical approach. [7]

In the second method, a best-fit cylinder to the SZ is computed to determine the axis. Best-fitting is performed using a least squares algorithm, and the screw axis is defined as the central axis of the resulting CYL. This is considered the most appropriate axis for a dorsal surgical approach[7].

In this study, most of the scaphoid waist projections appeared to be elliptical and backward-leaning. When K-wires are used to fix the scaphoid, the area of entry and distribution are important to avoid a cortical perforation. Based on the morphological characteristics of the axial view, we suggest that, in clinical practice, the K-wires be located in the RV quadrant because this area has the smallest perforation risk.

### RZs in the scaphoid

Iatrogenic perforation of the scaphoid may lead to non-union when the main artery is damaged. The dorsal artery supplies 70–80% of the blood to the scaphoid and transports away from the back side of the scaphoid waist[15]. The dorsal scaphoid branch is the only blood supply to the proximal pole. Most of the vascular foramina are also located within the UD and RD quadrants. Therefore, a screw (or K-wire) that perforates the UD or RD cortex may lead to a poor prognosis. According to our study, mean perforation risks were 27% (36%) and 18% (29%) with the volar and dorsal approaches, respectively, using screws (K-wires). Perforating screws also breach the intact cortex and significantly decrease fixation stability. At the same time, a perforating screw may damage other carpal bones, also contributing to a poor prognosis. When surgeons replace the internal fixators, we suggest that they avoid locating the start points close to the UD quadrant in both views. At the same time, the dorsal approach should be promoted because of its larger SZ. In this article, we described eight RZs with respect to the volar and dorsal views. A start point close to the RV quadrant is believed to have a lower risk of perforation and to lead to an easier operation with both the volar and dorsal approaches.

In conclusion, a screw that has actually perforated the scaphoid waist cortex can leave a false impression of adequate placement on both AP and lateral radiographs. The risks of perforation were 36% and 29% with the volar and dorsal percutaneous approaches, respectively. Surgeons should be wary of screws that appear to be close to the cortex, particularly in the UD quadrant, on both sets of radiographs, because such screw/K-wires are likely to perforate the cortex. The dorsal percutaneous approach is the method of choice because of the larger SZ. The position of the internal fixator should be assessed using our diagram showing RZs. The vertical view of the narrow part of the scaphoid projection is of utmost importance during surgery.

## Supporting Information

S1 Fig The effect of with or without radial bulge of scaphoid on percentage of safe zone. (a)The safe zone (SZ) into the RV and RD quadrant by calculated include the radial bulge of the scaphoid. (b) Rotating field of view (45°). (c) Rotating field of view (90°). (d) The safe zone (SZ) into the RV and RD quadrant by calculated without the radial bulge of the scaphoid. (e) Rotating field of view (45°). (f) Rotating field of view (90°).
(TIF)

S2 Fig The different planes in wrist coordinate system. a: The wrist coordinate system; b: coronal plane and central axis, c: horizontal plane and central axis; d: sagittal plane and central axis.
(TIF)

S1 File The explanation for supporting figures. (DOCX)

S1 Text Program code 1. (TXT)

S2 Text Program code 2. (TXT)

S3 Text Table 1 Relevant data underlying the findings described in manuscript. (XLSX)

S4 Text Tables 2 & 3 Relevant data underlying the findings described in manuscript. (XLSX)
