# Supplementary figures and images for: The Scaphoid Safe Zone: A Radiographic Simulation Study to Prevent Cortical Perforation Arising from Different Views
Source: PLoS One. 2017 Jan 23;12(1):e0170677. doi: 10.1371/journal.pone.0170677 (PMC5256911; doi:10.1371/journal.pone.0170677)

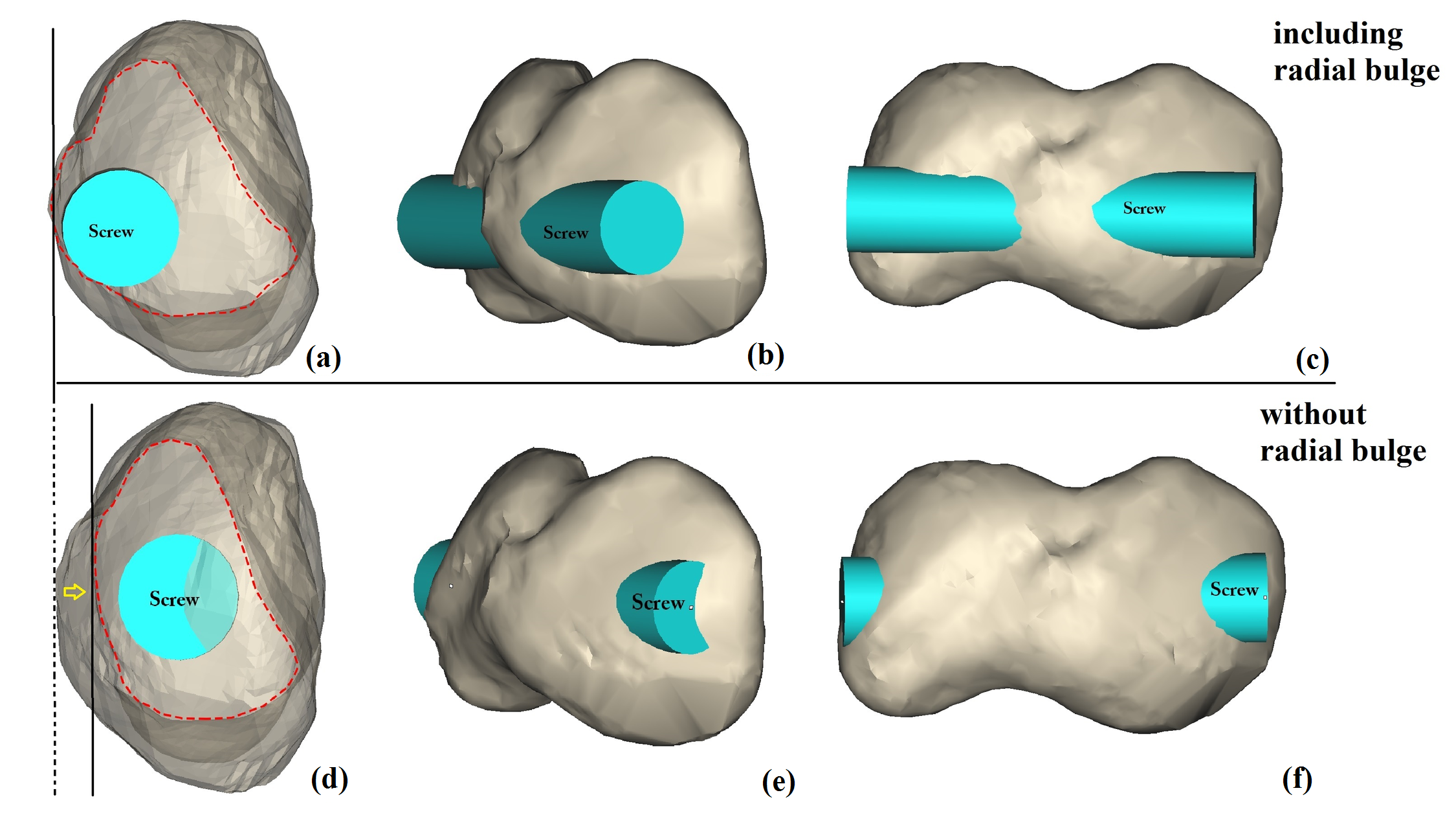

Supplement: S1 Fig — (a)The safe zone (SZ) into the RV and RD quadrant by calculated include the radial bulge of the scaphoid. (b) Rotating field of view (45°). (c) Rotating field of view (90°). (d) The safe zone (SZ) into the RV and RD quadrant by calculated without the radial bulge of the scaphoid. (e) Rotating field of view (45°). (f) Rotating field of view (90°). (TIF) [file pone.0170677.s001.tif]

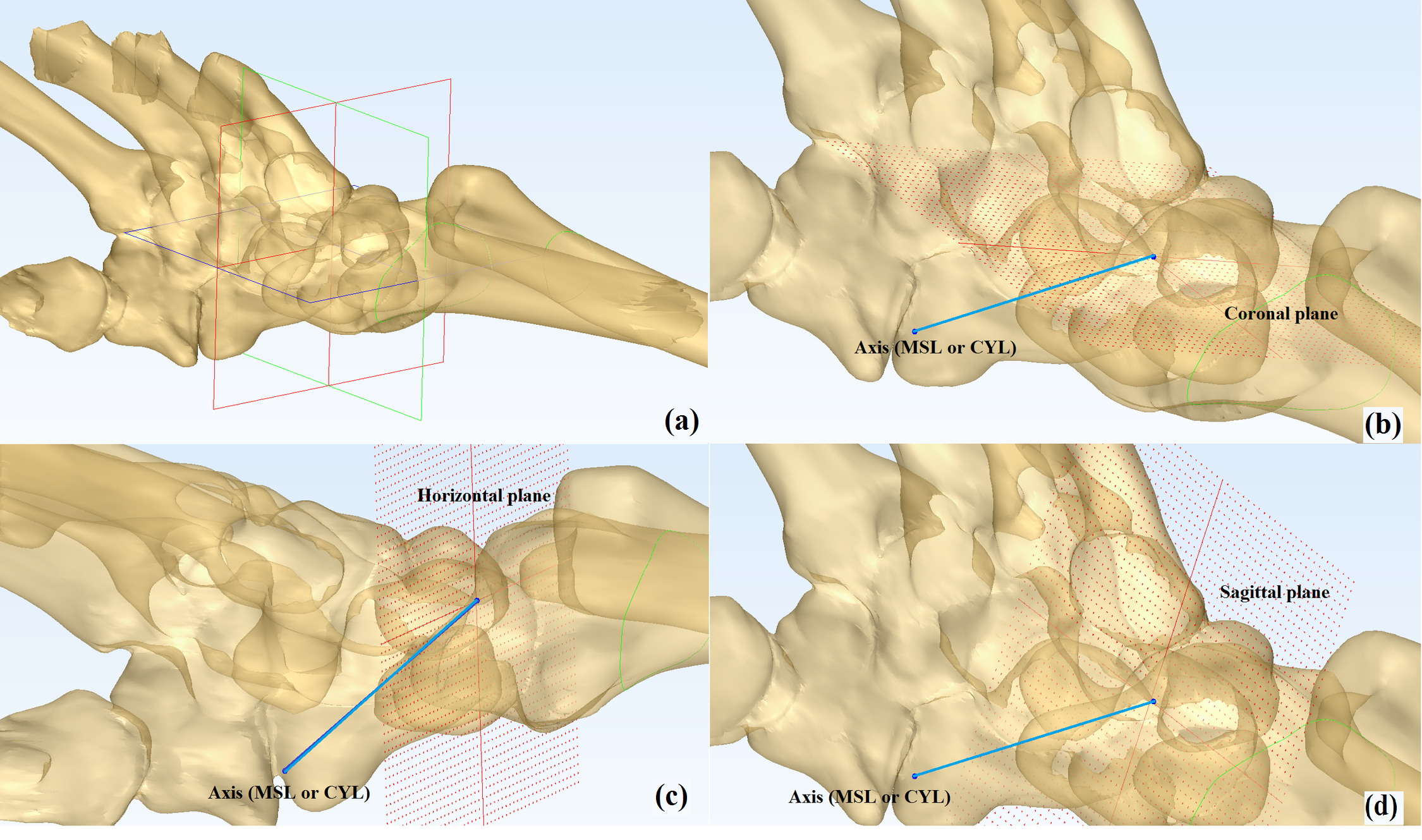

Supplement: S2 Fig — a: The wrist coordinate system; b: coronal plane and central axis, c: horizontal plane and central axis; d: sagittal plane and central axis. (TIF) [file pone.0170677.s002.tif]
