# Supplementary material for: The Scaphoid Safe Zone: A Radiographic Simulation Study to Prevent Cortical Perforation Arising from Different Views
Source: PLoS One. 2017 Jan 23;12(1):e0170677. doi: 10.1371/journal.pone.0170677 (PMC5256911; doi:10.1371/journal.pone.0170677)
Supplement: S1 File — (DOCX) [file pone.0170677.s003.docx]

**S1 Fig**


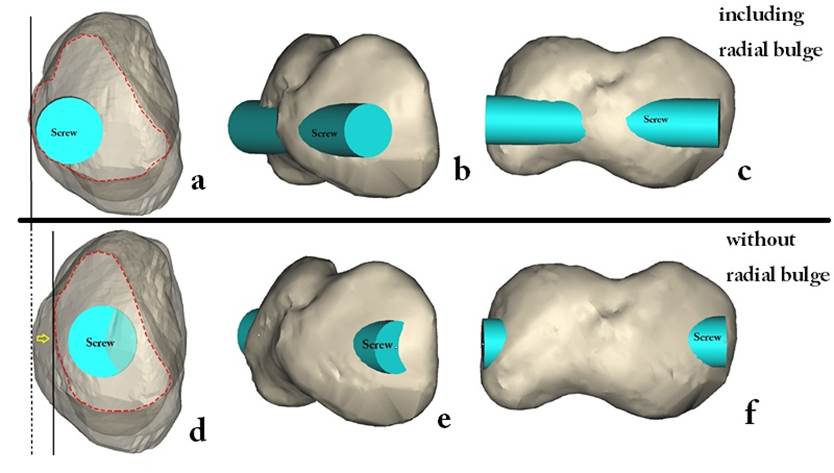


**S1 Fig. The effect of with or without radial bulge of scaphoid on percentage of safe zone.** (a)The safe zone (SZ) into the RV and RD quadrant by calculated include the radial bulge of the scaphoid. (b) Rotating field of view (45°). (c) Rotating field of view (90°). (d) The safe zone (SZ) into the RV and RD quadrant by calculated without the radial bulge of the scaphoid. (e) Rotating field of view (45°). (f) Rotating field of view (90°).

The safe zone (SZ) into the RV and RD quadrant by calculated include the radial bulge of the scaphoid (S1 Fig a). And make the results more accurate. However, the distance of screw travel is not too short (S1 Fig c). Although the screw was not piercing the cortex(S1 Fig a). But, due to the distance of screw travel is too short, fragment of fractures can not get a good fixation. The data is indeed more accurate by correcting for the radial bulge of scaphoid, but it increases the difficulty of the actual surgical application. Therefore, based on this consideration, we chose the most overlapping coordinates points of radial scaphoid cortical bone in axis cross-section for the radial side of the safe zone boundary. Although translating the radial border will reduce the percentage of safe zone. But, we believed that the results for inexperienced doctors have more surgical operation worth.

**S2 Fig**

**
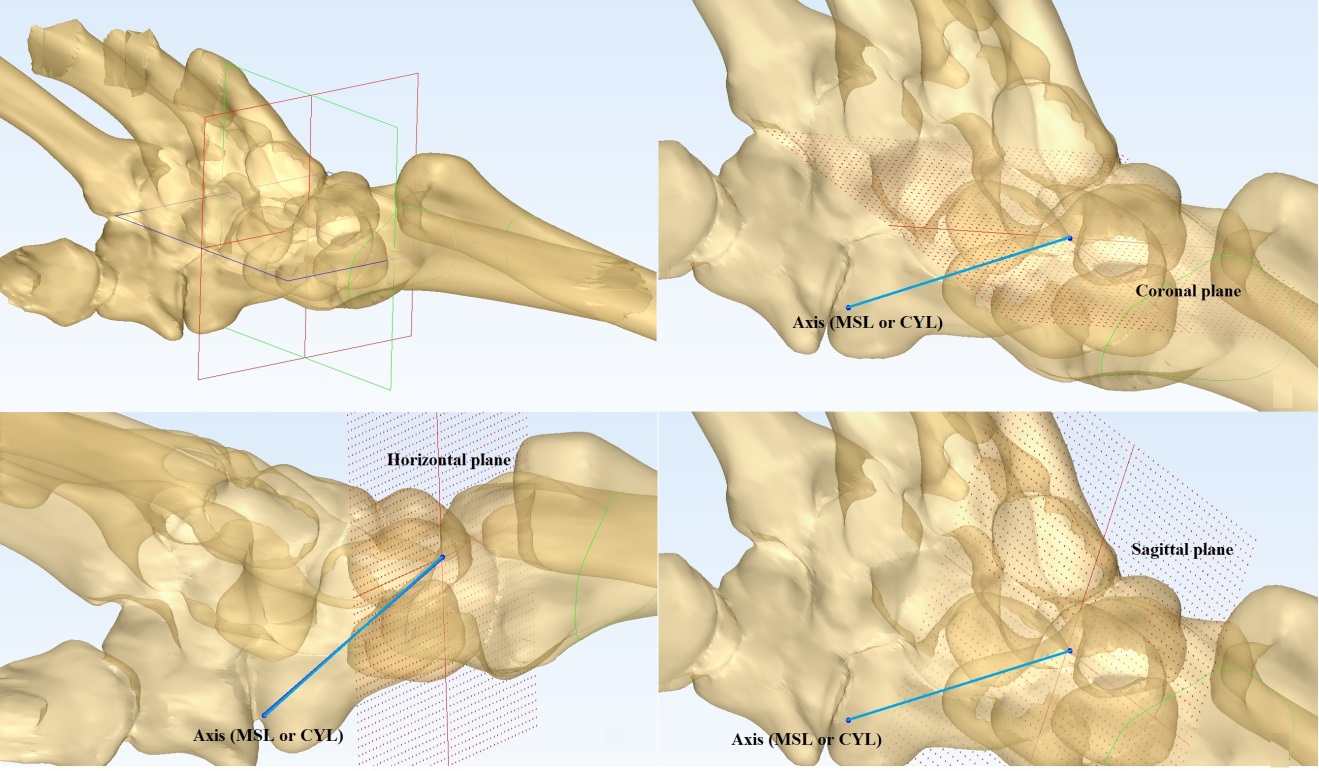
**

**S2 Fig. The different planes in wrist coordinate system.** a: The wrist coordinate system; b: coronal plane and central axis, c: horizontal plane and central axis; d: sagittal plane and central axis.
